# Smart Nanofibers with Natural Extracts Prevent Senescence Patterning in a Dynamic Cell Culture Model of Human Skin

**DOI:** 10.3390/cells9122530

**Published:** 2020-11-24

**Authors:** Emanuela Bellu, Giuseppe Garroni, Sara Cruciani, Francesca Balzano, Diletta Serra, Rosanna Satta, Maria Antonia Montesu, Angela Fadda, Maurizio Mulas, Giorgia Sarais, Pasquale Bandiera, Elena Torreggiani, Fernanda Martini, Mauro Tognon, Carlo Ventura, Jiří Beznoska, Evzen Amler, Margherita Maioli

**Affiliations:** 1 Department of Biomedical Sciences, University of Sassari, Viale San Pietro 43/B, 07100 Sassari, Italy; ema.bellu@hotmail.it (E.B.); giugarroni21@gmail.com (G.G.); sara.cruciani@outlook.com (S.C.); mariafrancesca22@virgilio.it (F.B.); dilettaserra9@gmail.com (D.S.); bandiera@uniss.it (P.B.); 2 Department of Medical, Surgical and Experimental Sciences, University of Sassari, 07100 Sassari, Italy; rsatta@uniss.it (R.S.); mmontesu@uniss.it (M.A.M.); 3 Istituto di Scienze delle Produzioni Alimentari (ISPA), Consiglio Nazionale delle Ricerche (CNR), Traversa la Crucca 3, 07100 Sassari, Italy; angela.fadda@cnr.it; 4 Department of Agriculture, University of Sassari, Via De Nicola 9, 07100 Sassari, Italy; mmulas@uniss.it; 5 Department of Life and Environmental Sciences, University of Cagliari, Via Ospedale 72, 09124 Cagliari, Italy; gsarais@unica.it; 6 Department Medical Sciences, Section Experimental Medicine, University of Ferrara, 44121 Ferrara, Italy; elena.torreggiani@unife.it (E.T.); fernanda.martini@unife.it (F.M.); mauro.tognon@unife.it (M.T.); 7 Laboratory of Molecular Biology and Stem Cell Engineering-Eldor Lab, National Institute of Biostructures and Biosystems, Innovation Accelerator, CNR, Via Piero Gobetti 101, 40129 Bologna, Italy; ventura.vid@gmail.com; 8 Institute of Biophysics, 2nd Faculty of Medicine, Charles University, V Uvalu 84, 150 06 Prague 5, Czech Republic; bezn@seznam.cz; 9 UCEEB, Czech Technical University, Trinecka 1024, 273 43 Bustehrad, Czech Republic; 10 Center for Developmental Biology and Reprogramming-CEDEBIOR, Department of Biomedical Sciences, University of Sassari, Viale San Pietro 43/B, 07100 Sassari, Italy; 11 Istituto di Ricerca Genetica e Biomedica, Consiglio Nazionale delle Ricerche (CNR), 09042 Monserrato, Italy

**Keywords:** stem cells, nanofibers, skin aging, cell senescence, 4D dynamic model, precision medicine, natural extracts, biophysics, cellular mechanisms

## Abstract

Natural cosmetic products have recently re-emerged as a novel tool able to counteract skin aging and skin related damages. In addition, recently achieved progress in nanomedicine opens a novel approach yielding from combination of modern nanotechnology with traditional treatment for innovative pharmacotherapeutics. In the present study, we investigated the antiaging effect of a pretreatment with *Myrtus communis* natural extract combined with a polycaprolactone nanofibrous scaffold (NanoPCL-M) on skin cell populations exposed to UV. We set up a novel model of skin on a bioreactor mimicking a crosstalk between keratinocytes, stem cells and fibroblasts, as in skin. Beta-galactosidase assay, indicating the amount of senescent cells, and viability assay, revealed that fibroblasts and stem cells pretreated with NanoPCL-M and then exposed to UV are superimposable to control cells, untreated and unexposed to UV damage. On the other hand, cells only exposed to UV stress, without NanoPCL-M pretreatment, exhibited a significantly higher yield of senescent elements. Keratinocyte-based 3D structures appeared disjointed after UV-stress, as compared to NanoPCL-M pretreated samples. Gene expression analysis performed on different senescence associated genes, revealed the activation of a molecular program of rejuvenation in stem cells pretreated with NanoPCL-M and then exposed to UV. Altogether, our results highlight a future translational application of NanoPCL-M to prevent skin aging.

## 1. Introduction

In the past few decades, aging-related diseases have emerged as major healthcare issues, due to prolonged lifespans [1]. Within this context, the need of a therapeutic intervention able to minimize age-related complications attracted the interest of pharmaceutical and cosmetic industries, with natural cosmetic products for the skincare attaining a growing interest [2,3].

Skin is a barrier showing many difficulties for transdermal delivery of active molecules since the stratus corneus affects the permeation of the right amount across the skin layers. Therefore, optimizing local drug delivery represents an important challenge in cosmesis [4].

The advent of nanomedicine opens up novel chances for innovative pharmacotherapeutic approaches [5]. Nanofibers are particularly interesting for their multiskilled features. Clearly, nanofibers are biomimetic, resembling the extracellular matrix, and they have broadly proved to stimulate tissue regeneration [6]. Nanofibers are also known as an excellent antimicrobial skin protecting cover. Unlike nanoparticles, nanofibers could be prepared as fully safe tools conforming all safety regulations for medical application. Nanofibers used in this study have been structurally designed to meet all restrictions for application in human medicine.

Nanofibers, thanks to their high porosity structure, allow nutrients and gas exchange with the skin. Moreover, nanofibers afford protection of covered tissue against bacterial and viral infection. Nanofibers can also be prepared with different composition of their shell and core. Thus, smart nanofibers enable accommodation of bioactive compounds on their surface due to their high ratio between the surface and the volume, as well as in their core. In addition, encapsulated bioactive compounds can be released from the nanofibers namely by controlled regulation of nanofiber decay [7,8]. We have constructed a smart nanofiber system for prevention of aging process and age-related complications by combining biodegradable nanofibers with natural compounds, rich in bioactive molecules.

Within this context, the Mediterranean area is rich in plants frequently used in traditional medicine, as myrtle (*Myrtus communis* L.*)*, belonging to Myrtaceae family. Some parts of this bush as for example berries, leaves, brushwood, are widely used for wound healing or for their antiseptic properties [9] besides of their application in food industry [10]. In traditional medicine, leaf and fruit decoctions were used to wash wounds, for vaginal lavage, and to counteract respiratory diseases [11]. Other formulations were used for gastrointestinal disorders, inflammation, conjunctivitis, epistaxis, and skin diseases [12]. The wide use of *Myrtus communis* in popular tradition, considering also the phytochemical, pharmacological and toxicological properties of the plant, has attracted great interest of the cosmetic and pharmaceutical industry. The bush exhibits both in vitro and in vivo antimicrobial, antioxidant and anti-inflammatory proprieties [13,14,15,16,17,18]. In particular, we previously demonstrated that myrtle extract was able to counteract adipose derived stem cell senescence, by downregulating the expression of senescence associated genes, while enhancing pluripotency and telomerase expression. Moreover, potential effects against chronic and acute inflammation were studied in both ethanol and aqueous extracts and essential oils [19,20].

The aging process is particularly evident on skin, as it represents the external part of the organisms, mostly exposed to injuries and damaging agents [21]. Skin aging arises from two different mechanisms: intrinsic and extrinsic. The intrinsic one is due to the passage of time and involves all the tissues of our organism. The extrinsic aging also called photoaging, is caused by continuous exposure of the skin to environmental agents, especially UVB, the most dangerous sunlight component, able to pass through the epidermidis reaching the dermis, resulting in DNA damage and increased oxidative stress [22,23]. Therefore, extrinsic aging activates several signals resulting in a decreased collagen production and an increased amount of matrix metalloproteases, causing connective tissue degradation, accumulation of senescent cells and components and degraded elastic fibers [24]. The detectable effects include the presence of wrinkles, enhanced epidermal thickness, loss of tone and hyperpigmentation [25]. Mesenchymal stem cells (MSCs) represent undifferentiated cells able to replace damaged elements and restore tissue function [26]. They are located in all tissues, in special areas called niches. Particularly, skin stem cells are responsible for keratinocyte differentiation, but not only, being enrolled after a damage to restore tissue homeostasis. The role of stem cells in self-renewal and differentiation is regulated by the expression of the main stemness genes: octamer-binding transcription factor 4 (Oct-4), sex-determining region Y-box 2 (Sox-2) and homeobox protein NANOG. During aging, also stem cells gradually lose their differentiation potential, a feature defined as multipotency or plasticity. This event is molecularly detectable by an ongoing downregulation of the stemness genes [27,28]. Within this context, premature stem cell senescence could represent a primary cause of aging, because senescent stem cells become unable to replace damaged elements. Beside stemness genes, also Bmi1, belonging to the polycomb and trithorax families of chromatin remodelling genes, prevents premature senescence. It maintains the stem cell pool, downregulating genes involved in senescence and/or telomerase reverse transcriptase activity. Telomerase reverse transcriptase (TERT) is part of a specialized ribonucleoprotein involved in stabilizing telomeres preventing their shortening, after cell division, gradually occurring during aging. Telomerase is highly expressed in proliferating cells but dramatically downregulated after differentiation and during cell aging. [28,29].

Skin senescence, as well as stressful conditions like UV exposure, is also accomplished by a downregulation of hyaluronic synthase 2 (HAS2) gene expression leading to a reduced hyaluronic acid synthesis by MSCs. A specific role of hyaluronic acid in maintaining cell viability, in particular during adverse conditions, has been also suggested [30].

The main aim of the present paper was to find out whether myrtle extracts from seeds, carrying specific previously described features [15], in combination with a nanofibrous scaffold could prevent skin aging process, stimulating tissue regeneration and whether the smart nanofiber from polycaprolactone (PCL) can potentially be used as medical device to enhance the delivery of bioactive molecules from myrtle extracts (NanoPCL-M).

Development and optimization of a novel skin model was another goal of this study. We set up a bioreactor with different crosstalk among cell populations to mimic in vivo organization of skin. To assess the skin ageing, we analyzed the expression of cell cycle genes, matrix production genes and β-galactosidase senescence-associated activity in skin derived stem cells and fibroblasts, in different conditions. Morphological effects of NanoPCL-M treatment on keratinocyte features were also evaluated by the aid of electron microscopes SEM and TEM.

## 2. Materials and Methods

### 2.1. Nanofibers Electrospinning and Combination with Myrtus Communis Extracts

PCL nanofibers were fabricated by the use of Multispin (Nanuntio, Prague, Czech Republic). The solution for the electrospinning process contained 10% (*w/v*) of PCL (MW 40 000 Wako Chemicals GmbH, Neuss, Germany), dissolved in a mixture of chloroform and ethanol at a ratio of 10:1. Electrospinning was performed using a needleless wire electrode, the fibers produced were deposited on a nonwoven supporting textile (Spunbond, Pegas Textiles, Prague, Czech Republic). After fabrication, produced nanofiber towels were stored at room temperature, samples of each were collected, put on aluminum stubs and coated with a gold layer employing a Polaron sputter-coater (SC510, Polaron, Now Quorum Technologies Ltd., Lewes, UK). The samples treated with gold were studied with electron microscope Aqua SEM (Tescan, Brno-Kohoutovice, Czech Republic). To perform the experiments, nanofibers towels were sterilized, cut and the samples were soaked with the myrtle extracts from seeds, previously molecularly analyzed and described [15], and left to dry before use.

### 2.2. Evaluation of Release Kinetic (from PCL Nanofiber of Myrtle Extract)

To analyze the release of the extracts from the nanofibers, we performed a kinetics release curve: nanofibers were left in PBS and absorbance (405 nm) was measured every day for one week. Kinetic release parameters were calculated using a standard curve (*R*^2^ = 0.9987; data not shown).

### 2.3. Cell Isolation and Culturing

Human skin stem cells were obtained from biopsies of adult male and female patients after ethics committee approval (Ethical Clearance N. 0021565/2018, 22/03/2018-Commissione Etica CNR). They were isolated and cultured as previously described [31]. Human skin fibroblast 1 (HFF1) and Human skin keratinocytes (HACAT) were purchased from ATCC (Manassas, VA, USA) and cultured in a Dulbecco’s modified Eagle’s Medium (DMEM) low-glucose medium (Life Technologies, Carlsbad, CA, USA), supplemented with 10% fetal bovine serum (FBS Life Technologies), 2 mM l-glutamine (Euroclone, Milano, Italy) and 1% of penicillin/streptomycin (Euroclone)[32].

### 2.4. Setting Up of Bioreactor and Cell Culture Conditions

**Figure 1:**
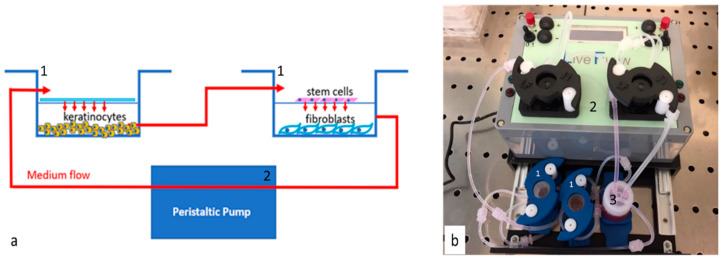
Scheme (**a**) and picture (**b**) of bioreactor. Panel a represent the chambers (Live Box2; blue lines (1)) and the cells cultured in every chamber. The chamber and the pump (Live Flow (2)) connections are visible by medium flow (red lines and arrows in the scheme). Panel b is the picture of the bioreactor used (IVTech) the chambers Live Box2 are blue (1); number (2) indicates the pump and number (3) the reservoir for the culture medium.

The bioreactor Live flow with the chambers Live Box2 (IVTech, 55054 Massarosa LU, Italy) was set up to be representative of the in vivo environment simulating the physiological conditions of skin layers. The bioreactor allows culturing different cell types separately, while maintaining the crosstalk thanks to the medium flow. Cells were counted with an automatic cell counter (LUNA, Logos biosystems, 11B avenue de l’Harmonie 59650 Villeneuve d’Ascq France), and seeded following the isometric proportion of the human skin tissue [33]: 40,000 Hacat, 20,000 HFF1 and 5000 stem cells. The first chamber, containing keratinocytes, is subsequently connected with the second one in which stem cells were seeded in the 0.45 µm membrane (ipCELLCULTURE™, it4ip, Avenue Jean-Etienne Lenoir 1, 1348 Louvain-la-Neuve, Belgium) fibroblasts in the glass below, and both are connected to reservoir and peristaltic pump as represented in the image (Figure 1).

NanoPCL-M was then inserted in the upper floor of the chamber containing keratinocytes. Flow rate was set at 0.1 mL/min for three and seven days. First, NanoPCL-M-treated cells (T) were exposed to oxidative stress, while control cells were kept in culture without any pretreatment (CS). Then, both pretreated (T) and non-pretreated cells (CS) were exposed to UV light for 2 min at 10 cm distance from the lamp to induce oxidative stress. Control untreated cells, not pretreated with NanoPCL-M, not stressed by UV, were cultured in the same conditions and used as negative control (C).

### 2.5. Evaluation of Cell Viability

The Thiazolyl Blue Tetrazolium Bromide assay (MTT) (Sigma-Aldrich, Saint Louis, MO, USA) was performed to evaluate the metabolic activity of HFF1 and skin stem cells. Cells were seeded at a concentration of 6000 cells/well in 96well plate pretreated with NanoPCL-M for three and seven days and then exposed to UV light as described before. After the culturing period of three or seven days in the conditions described above, MTT assay (Sigma-Aldrich) was performed. Cells belonging to all the different groups of treatment, were not confluent. Cell viability was detected by a plate reader (OD570) and expressed as percentage of cell viability referred to negative control (C). We also performed the MTT assay to evaluate the effect of PCL nanofiber alone, without myrtle extracts, on skin stem cells.

### 2.6. Senescence-Associated β-Galactosidase Staining

The ability of NanoPCL-M to prevent the aging process was evaluated by β-Galactosidase assay. Cells pretreated (T) or not (CS) with the Nano PCL-M for seven days, and then stressed by UV were stained with the Senescence-associated (SA) β-Galactosidase Staining Kit (Cell Signaling Technology, Euroclone, Milan Italy) according to the manufacturer protocol instructions. Qualitative detection of SA-β- Galactosidase activity, visible thanks to the blue staining, was detected under inverted microscope (Magnification 10X bright field). Cells not pretreated and not stressed (C) were used as negative control for senescence staining. Quantitative detection was analyzed with an image software analysis (ImageJ, version 1.8.0, National Institutes of Health, Bethesda, MD, USA).

### 2.7. Gene Expression of Stemness and Cell Cycle Genes

Gene expression levels were detected by Real Time-PCR after three and seven days of pretreatment with NanoPCL-M, as previously described. Total mRNA was isolated using RNeasy Mini Kit (Qiagen, 40724 Hilden, Germany) according to manufacturer protocol. Quantity and purity of RNA were measured by Od 260/280 nm using Nanodrop (Thermo Scientific, Waltham, MA, USA). RNA of each sample (2.5 ng) in triplicate was reverse-transcribed and amplificated with Power SYBR^®^ Green RNA-to-CT PCR under standard conditions [14] via the Thermal Cycler (Bio-Rad, Hercules, CA, USA). The qRT-PCR analysis was performed for the stemness markers Oct-4, Sox2 and Nanog, for the cell cycle related genes, p16 and p19, and for Bmi1, TERT and HAS2. All the primers were previously described [26,34]. Resulting Ct (Threshold Cycle) values normalization was performed on the housekeeping HPRT1 (Hypoxanthine Phosphoribosyltransferase 1) and mRNA levels were expressed as fold of change (2^−∆∆Ct^) as previously described [35] as compared to negative controls (C).

### 2.8. Electronic Microscope Morphological Analysis

Inside the chamber of Live Box2, keratinocytes organized themselves in a 3D structure. The fragments obtained were prepared for electron microscopy analysis. Samples were fixed with 2.5% glutaraldehyde solution in PBS for 1 h at 4 °C. The samples were then treated with 1% osmium tetroxide in PBS for 1 h at room temperature and then washed in PBS and stored at 4 °C. Samples were then dehydrated by passages in 50% and 100% acetone. Samples for SEM were dehydrated with hexamethyldisilazane and analyzed by scanning electron microscope (QUANTA 200, Fei, Thermo Scientific). Samples for TEM were included in epoxy resin (Durcupan^™^ ACM, Sigma-Aldrich) and then analyzed by Fei, TECNAI 20 (TECNAI 20, Fei, Thermo Scientific).

### 2.9. Statistical Analysis

All the experiments were performed in triplicate at the least two times (*n* = 10) and data were expressed as media ± standard deviation assuming statistically significant *p* value ≤ 0.05 (*).

## 3. Results

### 3.1. Nanofibers Features

**Figure 2:**
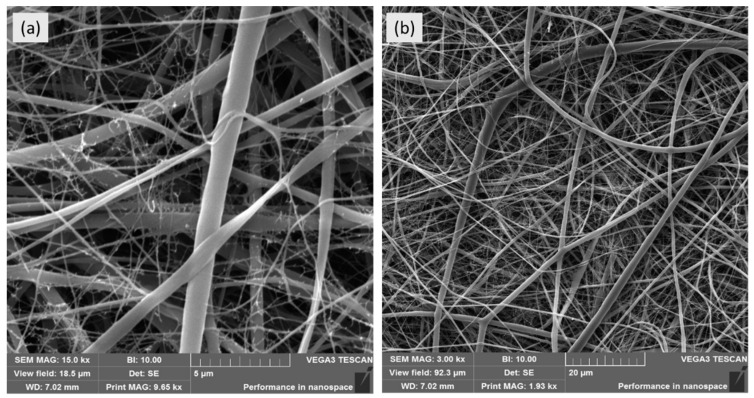
Images acquired with SEM microscope showing nanofiber structure. Panel (**a**) show nanofiber at 15 k magnification. Panel (**b**) show nanofiber at 3 k magnification.

PCL was used as a biodegradable polymer for nanofiber preparation by DC (direct current) spinning. The architecture of PCL nanofibers, investigated by scanning electron microscopy, showed a typical structure of PCL nanofibers with two peaks of the nanofiber diameter distribution at 250 nm and 2 µm, respectively (Figure 2).

### 3.2. NanoPCL-M Controls the Release of Myrtle Extracts

**Figure 3:**
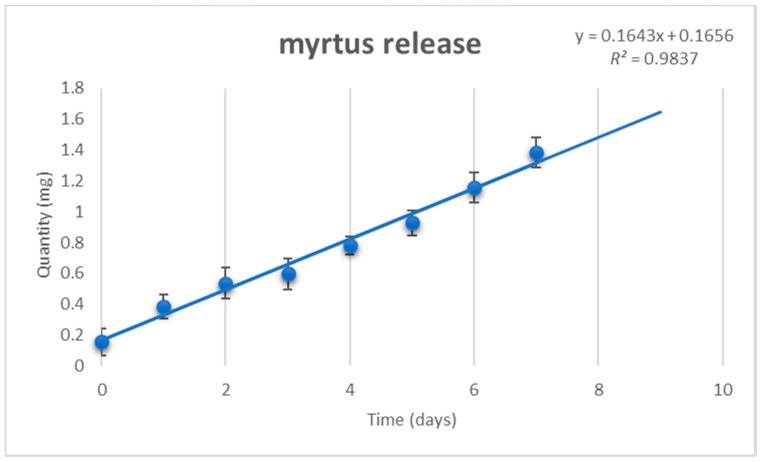
Release of myrtle extracts from NanoPCL-M for seven days. The amount of extracts is evaluated as absorbance OD detected at 405 nm, and expressed as mg/day. Error bars indicated standard deviation of the experiments (*n* = 6).

Spectrophotometry reveals the constant release of the myrtle extract from Nano PCL-M (Figure 3). The release allowed by the device was 0.22 ± 0.09 mg/day for seven days.

### 3.3. NanoPCL-M Pretreatment Preserves Cell Viability under UV Stress

**Figure 4:**
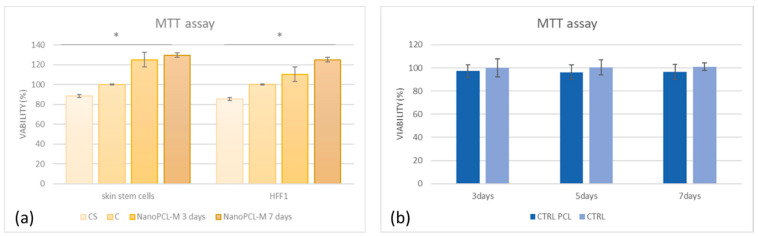
(**a**) The effect of NanoPCL-M on HFF1 fibroblasts and skin stem cells after three and seven days of pretreatment and then UV exposure. Graphs show the percentage of cellular metabolic activity as compared to negative control C (100%). CS represent cells untreated with NanoPCL-M and exposed to UV (stressed control), and C represents negative control. (**b**) effect of nanofiber in PCL (poly ε-caprolactone) (CTRL PCL) on stem cells compared with cells untreated (CTRL). Error bars represent standard deviation. * *p* value ≤ 0.05

NanoPCL-M pretreated (T) cells show higher proliferation as compared to control not-pretreated cells induced to senescence by UV (CS). Figure 4 panel a shows that, in control cells (both skin stem cells and HFF1) stressed by UV (CS), cell proliferation was lower than in control cells (C). Interestingly, NanoPCL-M pretreated cells reached a cell proliferation rate similar to control unstressed cells, being also significantly higher when cells were pretreated for seven days. The use of the nanofiber alone without myrtle extracts had no significant impact on cell viability (Figure 4 panel b).

### 3.4. NanoPCL-M is able to Counteract UV-Induced Cell Senescence

**Figure 5:**
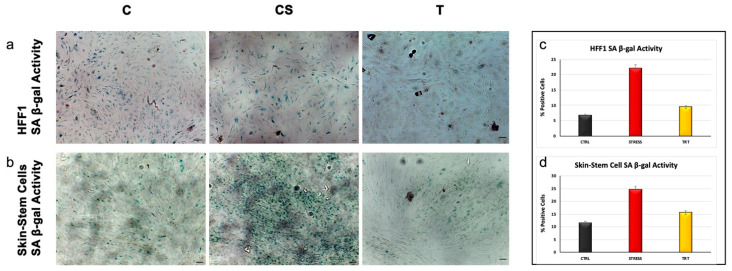
Senescence-associated β-galactosidase activity evaluated in HFF1 (**a**) and skin stem cells (**b**) after seven days. Cells pretreated with NanoPCL-M (T) are compared to control untreated cells (C) and UV stress control (CS). Scale bar = 100 µm. The number of blue positive HFF1 (**c**) and skin stem (**d**) was calculated using ImageJ. Data are expressed as mean ± SD.

β-galactosidase staining assay, performed on skin stem cells and fibroblast HFF1, revealed that UV treatment induced a significant increase in blue-stained senescent cells, as compared to control cells (C). Noteworthy Nano PCL-M pretreated samples exhibited a number of senescent cells almost superimposable to control unstressed cells(C), even after UV exposure (Figure 5).

### 3.5. NanoPCL-M Maintains a Molecular Program of Youngness

**Figure 6:**
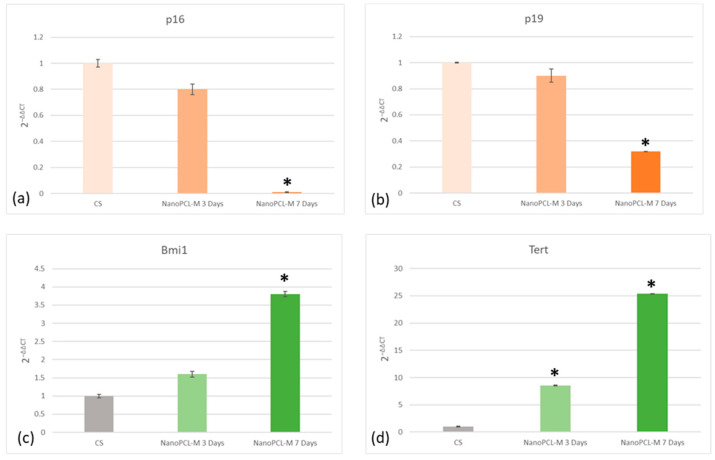
Effect of NanoPCL-M pretreatment on the expression of p16 (**a**), p19 (**b**), Bmi1 (**c**) and TERT (**d**). Skin stem cells exposed (T) or not (CS) to three to seven days of NanoPCL-M pretreatment were stressed by UV light. The amount of mRNA from NanoPCL-M treated cells was normalized to HPRT1 and was plotted as fold change (2^−∆∆CT^) relative to the mRNA expression of UV stress control (CS). * *p* value ≤ 0.05.

**Figure 7:**
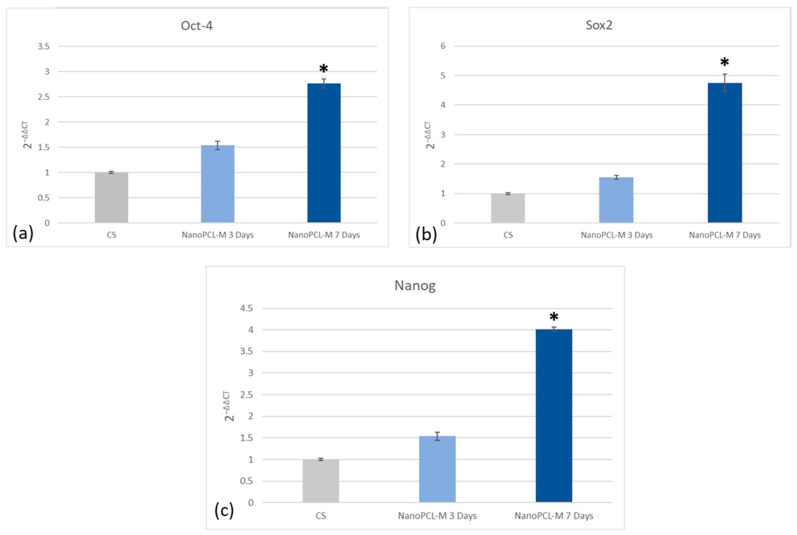
Effect of NanoPCL-M pretreatment on the expression of Oct-4 (**a**), Sox2 (**b**) and Nanog (**c**). Skin stem cells exposed (T) or not (CS) to three to seven days of NanoPCL-M pretreatment were stressed by UV light. The amount of mRNA from NanoPCL-M treated cells was normalized to HPRT1 and was plotted as fold change (2^−∆∆CT^) relative to the mRNA expression of UV stress control (CS). * *p* value ≤ 0.05

**Figure 8:**
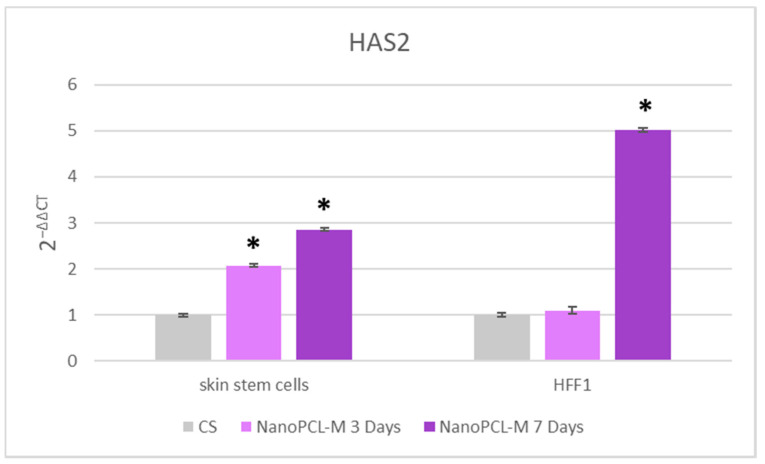
Effect of NanoPCL-M pretreatment on the expression of HAS2. Skin stem cells and HFF1 exposed (T) or not (CS) to three to seven days of NanoPCL-M pretreatment were stressed by UV light. The amount of mRNA from NanoPCL-M treated cells was normalized to HPRT1 and was plotted as fold change (2^−∆∆CT^) relative to the mRNA expression of UV stress control (CS). * *p* value ≤ 0.05

Real time qPCR reveals that pretreatment with NanoPCL-M before UV exposure was able to prevent the UV-induced downregulation of TERT and Bmi1 gene expression in skin stem cells (Figure 6). Concomitantly, the same pretreatment inhibited the appearance of the senescence-regulating genes p16 and p19. Moreover, the expression of the stemness genes Oct-4, Sox2 and Nanog was significantly reduced in UV-treated cells (CS), as compared to NanoPCL-M pretreated cells (T) exposed to UV stress (Figure 7). NanoPCL-M pretreatment was also able to induce the overexpression of HAS2 gene, related to extracellular matrix production, in both HFF1 and stem cells along with all the culturing period (Figure 8).

### 3.6. NanoPCL-M Pretreatment Preserve Keratinocytes’ Main Features also after Stress

**Figure 9:**
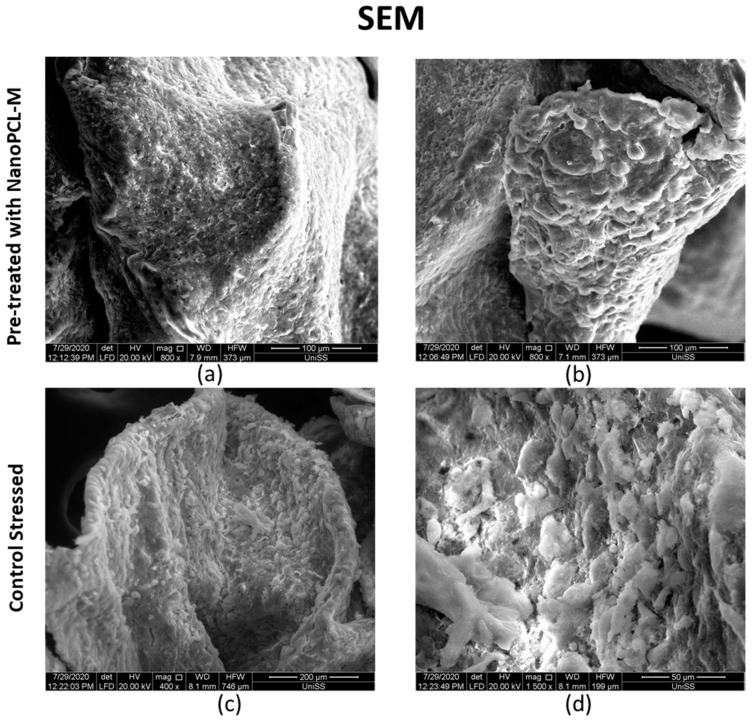
Images acquired with SEM microscope showing keratinocytes 3D organization. (**a**,**b**) represent UV stressed samples pretreated (T) with NanoPCL-M for seven days. (**c**,**d**) represent UV stressed samples (CS) kept in culture for seven days without any pretreatment. Scale bar is indicated in each panel.

SEM morphological analysis highlighted the presence of a polarity in the fragments observed with two different surfaces (Figure 9). One surface (a,c), more compact and homogeneous, was identified as the one in direct contact with the slide. The other surface (b,d) was irregular: cellular elements of the most superficial layer were not in contact to each other, suggesting a proliferative process, in which cells seem to migrate toward the most superficial portion of the sample. Nevertheless, SEM analysis also showed a difference in the morphology of cells located on the surface (b,d) between the analyzed samples. Stressed control cells (CS) exhibited UV induced damages with keratinocytes located in the surface being flattened, not well distributed, with poorly defined profiles. On the other hand, keratinocytes of the NanoPCL-M pretreated sample (T) and of the negative control (C) (data not show) were rounded and well defined and morphologically similar, and it was impossible to identify significant differences (Figure 9).

**Figure 10:**
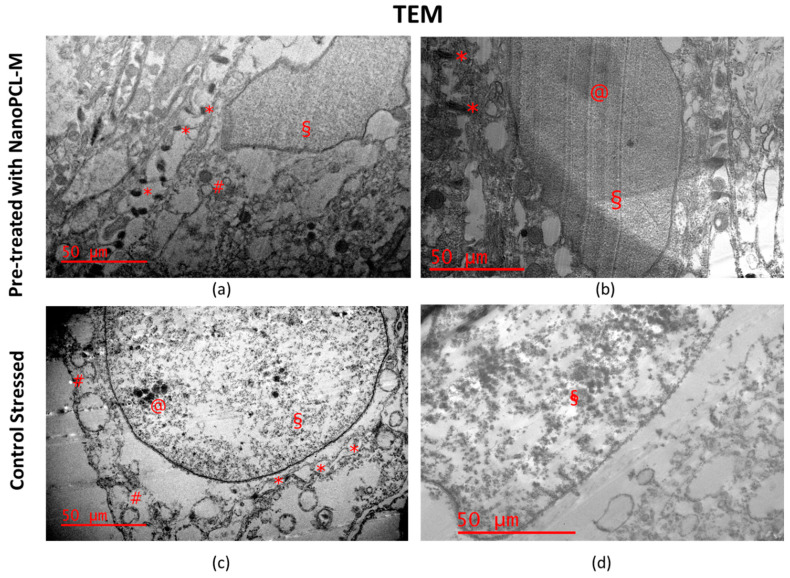
Images acquired with TEM showing keratinocytes 3D organization. Scale bar = 50 um. (**a**,**b**) represent UV stressed samples pretreated (T) with NanoPCL-M for seven days. (**c**), (**d**) represent UV stressed samples (CS) kept in culture for seven days without any pretreatment. * Desmosomes; **§** Chromatin; **#** Vacuoles. Desmosome junctions (*) appeared regular and preserved intracellular junctions in T samples ((**a**,**b**)*); on the contrary they were altered and degenerated in morphology in CS samples (panel c *). Chromatin (**§)** was homogenous like euchromatin in the T sample ((**a**,**b**) *) with visible nucleolus ((**b**) **@**). On the other hand chromatin (**§)** of CS samples appeared disorganized, not homogenous ((**c**,**d**) **§**) and fragmented ((**d**) **§**). Vacuoles shows irregular membrane in CS samples ((**c**) #) and are present in low number and smaller in T samples ((a) #).

TEM analysis showed significant differences between control cells under UV stress (CS) (Figure 10 panels c,d) and in samples pretreated with NanoPCL-M (T) (Figure 10 panels a,b). Membranes of CS keratinocytes were damaged, and cells lost their adhesion properties probably due to cytoskeleton disarrangement. Moreover, cellular organelles were also damaged, the cytoplasm was disorganized and the nucleus appeared inhomogeneous with wide areas of fragmented heterochromatin. Noteworthy, a large number of vacuoles with damaged membranes could be observed. Indeed, keratinocytes pretreated with NanoPCL-M preserved cellular integrity: desmosomes’ junction structure wax distinguishable and both nucleus and membranes appeared regular. Chromatin looked homogeneous with a well-defined nucleolus. Nevertheless, vacuoles are present even though they were fewer, showing a defined membrane, as compared to those observed in CS keratinocytes.

## 4. Discussion

In the skin, the fundamental resource for self-renewal is represented by skin stem cells, which, as well as stem cells derived from other tissues, are able to differentiate towards a selected cell phenotype, under specific stimuli [31,35,36,37]. Indeed, skin stem cells are closely related to the activity of the other cell types resident in the tissue. The use of a bioreactor allows to sustain cellular crosstalk typical of native microenvironment. Furthermore, fluidic systems propagate the medium in the chambers, while keeping the isolated culture of each cell type [38]. Here, the bioreactor was applied for culturing keratinocytes, stem cells, and fibroblasts, creating a 3D keratinocyte organization, thus resembling the superficial layer of the skin. Moreover, the presence of the flow creates a microenvironment where cell communication happens. The dynamicity generated by medium flow represents the fourth dimension of our system, combining 3D organization and cellular communication. Skin regeneration is a complex process in which skin fibroblasts and stem cells play a crucial role, replacing damaged cellular elements and producing extracellular matrix and hyaluronic acid secretion [39]. Within this context, plants, herb extracts and other bioactive molecules are actually applied as active drugs for tissue regeneration. They are commonly used in traditional medicine to treat skin diseases, even for their antiaging and photoprotective effect [15,19].

In the present study, we applied nanotechnologies such as PCL nanofibers to allow a controlled release of *Myrtus communis* extract. The delivered pretreatment with NanoPCL-M vehiculated in the cultures by the flow of bioreactor, mimics a topic treatment that reaches first keratinocytes, then the basal layer containing stem cells, and ultimately the dermis, mainly comprised by fibroblasts.

Here, we show that these functionalized nanofibers (NanoPCL-M) were able to increase stem cell and fibroblasts viability, counteracting aging triggered by oxidative stress. Moreover, the cell senescence test (β-galactosidase), performed on fibroblasts and stem cells, highlights the protective effect of Nano PCL-M on aging induced by UV irradiation. Nevertheless, a molecular program of senescence, usually activated after UV stress is blocked by NanoPCL-M pretreatment. p16 and p19 genes are usually induced when cells are damaged, or after prolonged division, to block cell cycle [26,36]. In particular, p16 inhibits the transition from G1 to S-phase in cell cycle progression [39], exhibiting a fundamental role in homeostasis and aging, maintaining undifferentiated keratinocytes in the deep layers of the tissue. In the skin basal layer, epigenetic modifications repress p16 expression, protecting stem cells from senescence and preserving the regeneration capability of the tissue [39]. Interestingly, in our experiments we could detect a downregulation in the expression of both these senescent associated genes in stem cells pretreated with NanoPCL-M and then exposed to UV stress, as compared to cells only exposed to UV. These results highlight a protective effect of our nanodevices, able to save cells from the cell cycle exit and then from a senescence fate. Stem cell plasticity and regeneration capability is highly dependent on the expression of the stemness related genes as Oct-4, Sox-2 and Nanog, whose activity is gradually reduced during the senescence process, thus counteracting regeneration of damaged tissue [37,39]. Nevertheless, it has been proved that MSCs could undergo premature cellular senescence with a defective telomerase activity, associated with a progressive decline in the expression of mesenchymal stem cell markers [40,41]. Within this context, Bmi1 is transcriptionally downregulated when cells undergo replicative senescence [42], thus allowing the expression of p16 and p19. Interestingly, Bmi1 is also able to modulate stemness genes as Oct 4 and Sox2 [39]. Here, we found that NanoPCL-M not only increased the transcription of Bmi1, Oct4, Sox2, Nanog, but it also behaved as an important positive regulator of the TERT gene, thus counteracting telomere shorting.

Another component of the skin deeply involved in senescence is the cellular matrix, a main constituent of dermis [22,23]. HAS2 gene, coding for hyaluronan synthase, and expressed both in fibroblasts and in stem cells, is involved in matrix production, thus being a good indicator of the normal physiologic properties of the skin. HAS2 is the isoform of hyaluronan synthase most responsible for hyaluronan production in mesenchymal cells and it has been implicated in cellular senescence, since its suppression causes the arrest of cells in the G1 phase [43]. The synthesis of hyaluronic acid has a crucial role in preserving cell viability under stressing stimuli, also regulating apoptosis in skin fibroblasts [44]. In the present study, we demonstrate the capability of NanoPCL-M to induce HAS2 gene expression, both in HFF1 and stem cells, suggesting a possible enhancement of extracellular matrix production. According to these results, a potential role of our nanodevice in maintaining a molecular program, encompassing the features of young dermis, could be hypothesized. Other authors previously described the role of UVA irradiation in interfering with different processes crucial for skin homeostasis, including, adhesion, proliferation, differentiation, senescence, neoplastic transformation and cell death, accomplished by detectable changes in cellular features [30]. Here, we highlight the effect of NanoPCL-M in preventing morphological changes associated with aging in keratinocyte populations organized in the 3D structure that appeared significantly less damaged than in the samples exposed to UV only. Overall, our results indicate that NanoPCL-M is able to interfere with the molecular mechanisms responsible for aging induced by UV, involving the pathway of cell pluripotency and cellular senescence.

## 5. Conclusions

In the present paper, we highlight that functionalized PCL nanofibers could be successfully applied in cosmetic products for their physiochemical properties, able to release under control bioactive substances and, thus, prevent the aging process in a skin dynamic model. [45]. Electrospun smart nanofibers could represent an attractive alternative to classical commercial masks, as for example nanofibers to vehiculate collagen, ascorbic acid, hyaluronic acid and other molecules on skin [46,47]. Our NanoPCL-M, based on a biocompatible and human-health-safe PCL molecule and a natural compound, can potentially be used also as medical device, thus being a perfect candidate for topic application.
