# Correction: Bellu et al. Smart Nanofibers with Natural Extracts Prevent Senescence Patterning in a Dynamic Cell Culture Model of Human Skin. *Cells* 2020, *9*, 2530

**DOI:** 10.3390/cells13151285

**Published:** 2024-07-31

**Authors:** Emanuela Bellu, Giuseppe Garroni, Sara Cruciani, Francesca Balzano, Diletta Serra, Rosanna Satta, Maria Antonia Montesu, Angela Fadda, Maurizio Mulas, Giorgia Sarais, Pasquale Bandiera, Elena Torreggiani, Fernanda Martini, Mauro Tognon, Carlo Ventura, Jiří Beznoska, Evzen Amler, Margherita Maioli

**Affiliations:** 1 Department of Biomedical Sciences, University of Sassari, Viale San Pietro 43/B, 07100 Sassari, Italy; ema.bellu@hotmail.it (E.B.); giugarroni21@gmail.com (G.G.); sara.cruciani@outlook.com (S.C.); mariafrancesca22@virgilio.it (F.B.); dilettaserra9@gmail.com (D.S.); bandiera@uniss.it (P.B.); 2 Department of Medical, Surgical and Experimental Sciences, University of Sassari, 07100 Sassari, Italy; rsatta@uniss.it (R.S.); mmontesu@uniss.it (M.A.M.); 3 Istituto di Scienze delle Produzioni Alimentari (ISPA), Consiglio Nazionale delle Ricerche (CNR), Traversa la Crucca 3, 07100 Sassari, Italy; angela.fadda@cnr.it; 4 Department of Agriculture, University of Sassari, Via De Nicola 9, 07100 Sassari, Italy; mmulas@uniss.it; 5 Department of Life and Environmental Sciences, University of Cagliari, Via Ospedale 72, 09124 Cagliari, Italy; gsarais@unica.it; 6 Department Medical Sciences, Section Experimental Medicine, University of Ferrara, 44121 Ferrara, Italy; elena.torreggiani@unife.it (E.T.); fernanda.martini@unife.it (F.M.); mauro.tognon@unife.it (M.T.); 7 Laboratory of Molecular Biology and Stem Cell Engineering-Eldor Lab, National Institute of Biostructures and Biosystems, Innovation Accelerator, CNR, Via Piero Gobetti 101, 40129 Bologna, Italy; ventura.vid@gmail.com; 8 Institute of Biophysics, 2nd Faculty of Medicine, Charles University, V Uvalu 84, 150 06 Prague 5, Czech Republic; bezn@seznam.cz; 9 UCEEB, Czech Technical University, Trinecka 1024, 273 43 Bustehrad, Czech Republic; 10 Center for Developmental Biology and Reprogramming-CEDEBIOR, Department of Biomedical Sciences, University of Sassari, Viale San Pietro 43/B, 07100 Sassari, Italy; 11 Istituto di Ricerca Genetica e Biomedica, Consiglio Nazionale delle Ricerche (CNR), 09042 Monserrato, Italy

**Error in Figure**

In the original publication [1], there was a mistake in Figure 5 as published. We mistakenly incorporated four incorrect panels into Figure 5 due to their high similarity with images obtained from our previous experiments with cells cultured under control conditions and exposed to UV stress. We apologize for this inconvenience. The corrected Figure 5 appears below. The authors state that the scientific conclusions are unaffected. This correction was approved by the Academic Editor. The original publication has also been updated.

## Figures and Tables

**Figure 5:**
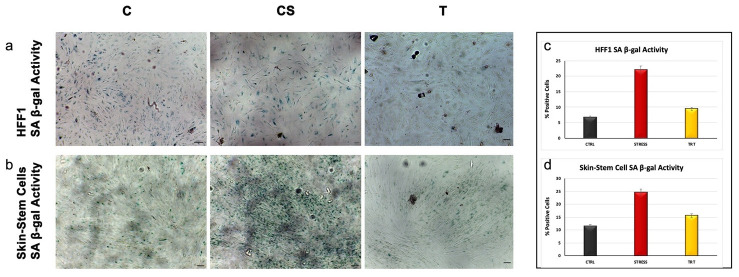
Senescence-associated β-galactosidase activity evaluated in HFF1 (**a**) and skin stem cells (**b**) after seven days. Cells pretreated with NanoPCL-M (T) are compared to control untreated cells (C) and UV stress control (CS). Scale bar = 100 µm. The number of blue positive HFF1 (**c**) and skin stem (**d**) was calculated using ImageJ. Data are expressed as mean ± SD.
